# Supplementary material for: Accurate Screening for Early-Stage Breast Cancer by Detection and Profiling of Circulating Tumor Cells
Source: Cancers (Basel). 2022 Jul 9;14(14):3341. doi: 10.3390/cancers14143341 (PMC9316476; doi:10.3390/cancers14143341)
Supplement: Supplementary file 1 [file cancers-14-03341-s001.zip › cancers-1758592-supplementary.pdf]

File S1: Supplementary Materials, Methods and Findings

Markers

The test identifies breast adenocarcinoma associated circulating tumor cells (BrAD-CTCs) as malignant apoptosis resistant cells positive for EpCAM, PanCK, GATA3 and GCDFP-15 and negative for CD45. Pan-Cytokeratins (PanCK) are a family of cytoplasmic structural proteins that are present in epithelial tumors and in CTCs. PanCK positivity along with absence of CD45 expression is the primary determinant of CTCs. Epithelial Cell Adhesion Molecule (EpCAM) are membrane antigens present on epithelial cells (and carcinomas) that function in cell adhesion. GATA Binding Protein 3 (GATA3) is a transcription factor (nuclear marker) which is commonly overexpressed in breast (up to 94%), salivary duct, skin and urothelial carcinomas, and less commonly in carcinomas of the lungs, liver, pancreas, stomach, kidneys, thyroid, endometrium and ovary. Gross Cystic Disease Fluid Protein 15 (GCDFP15) expression is commonly reported in breast (up to 90%) and salivary duct carcinomas, and less commonly in carcinomas of the lungs and prostate. Common Leucocyte Antigen (CD45) serves to discern the CTCs from CD45 positive haematolymphoid cells. The combination of GATA3 and GCDFP15 ensures a high specificity of organ localisation. Classically, this combination of markers is used to identify a Breast primary in case of metastatic progression or recurrence. Neither marker is reported to be expressed in normal breast parenchyma or non-malignant conditions of breasts. Co-expression of GATA3 and GCDFP15 is reported in salivary duct carcinoma (SDC), a rare cancer type with significantly lower ($1/200^{th}$) incidence than breast cancer.

Antisera and Cell Lines

The antisera used included recombinant human (RH) Anti CD326 IgG1-Vio 615 (Miltenyi Biotech, Clone REA764 (#130-111-006)), RH Anti-CK-IgG1-Vio 515 (Miltenyi Biotech, Clone REA831 (#130-112-746)), RH Anti-CD45-IgG1-APCVio 770 (Miltenyi Biotech, Clone REA747 (#130-110-635)), Mouse Anti-GATA3 IgG1/k (Dako, Clone L50-823 (#MAD-000632QD-R-3)), Mouse Anti-GCDFP15 IgG1/k (Dako, Clone 23A3 (#BSB 5555)) and Anti-Mouse Alexa Fluor 594 (Invitrogen (#A32742)). The reference cell lines include SKBR3 (breast cancer), MOLT-3 (leukemia) and SW982 (synovial sarcoma) all of which were procured from ATCC. The purity of reference cell lines was confirmed by Short Tandem Repeat (STR) Profiling and testing for Mycoplasma every 6 months.

Isolation of Primary Tumor Derived Cells

The isolation of primary tumor derived cells (TDCs) from an excised tumor (malignant / benign) has been described previously [1].

Enrichment of Circulating Tumor Cells from Peripheral Blood

Aliquoted blood samples (5 mL) were processed for the enrichment of circulating tumor cells (CTCs) from peripheral blood mononuclear cells (PBMC) as described previously [1]. Briefly, PBMCs were isolated from whole blood via lysis of red blood cells (RBCs) followed by centrifugation. PBMCs resuspended in Phosphate Buffered Saline (PBS) were treated with a proprietary differentially cytotoxic medium that induces cell death in all apoptosis-competent non-malignant (hemato-lymphoid, epithelial and endothelial) cells, while malignant tumor derived cells (CTCs) survive due to apoptosis resistance. After treatment for 5 days at 37°C, surviving cells and cell clusters are harvested by centrifugation and resuspended in PBS.

Immunocytochemistry Profiling of Circulating Tumor Cells

The process of ICC profiling of CTCs has been described previously [2]. Briefly, CTCs enriched from 5 mL of blood were resuspended in 1500 μL 1x Phosphate Buffered Saline (PBS) and 100 μL aliquots of enriched CTCs seeded into 15 wells. Cells in each well were equivalent to 333 μL blood sample. Cells were fixed with 4% Paraformaldehyde, permeabilized with 0.3% Triton-X 100 and treated with 3% BSA (blocking). Cells were immunostained with each of the 3 separate Primary (1°) Ab cocktails for multiplexed analysis of the following combination of markers, (a) Anti-PanCK (1:500), Anti-CD45 (1:500), Anti-EpCAM (1:500), (b) Anti-PanCK (1:500), Anti-CD45 (1:500), Anti-GATA3 (1:4), (c) Anti-PanCK (1:500), Anti-CD45 (1:500), Anti-GCDFP15 (1:2). Samples for GATA3 and GCDFP15 were incubated with secondary (2°) anti-mouse Ab (1:500). PBS washes followed each Ab incubation step. Each marker combination was evaluated in 5 wells (333 μL × 5 = 1.67 mL equivalent of blood). Finally, cells were treated with 4',6-Diamidino-2-phenylindole dihydrochloride (DAPI) for nuclear staining. Control samples (SKBR3 for EpCAM, GATA3 and GCDFP15 and MOLT3 for CD45) were included in each run. Samples were evaluated on the CellInsight High Content Screening (HCS) Platform to determine the Fluorescence Intensity (FI) for each marker. Marker expression was determined by the sequential excitation and acquisition of fluorescence signal.

Cells isolated from a primary benign or malignant tumor were resuspended in phosphate buffered saline (PBS) solution and ICC profiled similarly as described above for CTCs.

Method Development

Detection Thresholds

SKBR3 cells, MOLT-3 cells, SW982 cells, BrAD-CTCs, malignant breast tumor derived cells (M-TDCs) and benign breast tumor derived cells (B-TDCs) were immunostained for all markers (20 replicates). FI was recorded for each marker to determine relative expression of each marker per cell type.

Finding: The FI of PanCK, GATA3, GCDFP15 and EpCAM were higher in SKBR3, BrC-TDC (M-TDC) and BrAD-CTCs than in MOLT-3, SW982 and benign breast tumor cells (B-TDC). The FI of CD45 was significantly higher in MOLT-3 than in the other cell types. FI of markers was lowest in SW982 (Supplementary Figure S2). Based on these findings, the FI threshold for positivity was assigned as 70,000 (relative fluorescence units, RFU) for PanCK and EpCAM and 50,000 RFU for GATA3 and GCDFP15; these apply as a lower threshold for SKBR3 (positive control (PC) for all 4 markers), BrC-TDC and BrAD-CTCs where expression is essential for positivity. These FI thresholds accommodate CTCs with lower marker expression than the M-TDC or cell lines, such as CTCs undergoing epithelial to mesenchymal transition (EMT). For CD45 40,000 RFU was set as the upper threshold in SKBR3, M-TDC and BrAD-CTCs where expression is not expected and as the lower threshold for MOLT-3 (PC for CD45, negative control (NC) for all other markers). Additionally, numerical thresholds were defined as the proportion of cells staining positively for each marker for the acceptance of positive control (PC; >60%) and negative control (NC; <1%).

Marker Specificity

We determined the specificity of the marker combination to BrC by evaluating their expression in various CTCs. FI for GATA3 and GCDFP15 were evaluated after

immunostaining of CTCs from Cancers of the Cervix, Esophagus, Kidney, Lung, Ovary, Pancreas and Stomach.

Finding: As expected, expression of GATA3 and GCDFP15 was lower (FI < 50,000 U) in all non-BrC CTCs (Supplementary Figure S3).

Marker Expression in Breast Cancer

FI for GATA3, GCDFP15, EpCAM, PanCK and CD45 were evaluated in subsets of BrAD-CTCs stratified by Age-Group (n=249), Ductal v/s Lobular subtype (n=219), Grade (n=99), Hormone Receptor Status (n=159) and Stage (n=162). FI of all markers was also evaluated in BrAD-CTCs from a Caucasian population (n=65) and from a South Asian population (n=225) to determine if there are any variances due to ethnicity.

Finding: As can be seen in Supplementary Figures S4 – S8, there were no significant variations in FI of any marker due to any of these intrinsic factors. Similarly, expression of markers in CTCs from Caucasian samples were compatible with (not lower than) the FI thresholds which were established in CTCs from South Asian samples (Supplementary Figure S9).

BrAD-CTCs in Benign or Inflammatory Breast Conditions

To determine the specificity of the Test to discern BrC from non-malignant conditions of the Breast, we evaluated blood samples from 91 recently diagnosed cases of benign or inflammatory conditions of the breast (Supplementary Table S10). Samples were processed for CTC enrichment and ICC profiling as described above.

Finding: BrAD-CTCs were not detected in any cases indicating a specificity of 100% (cancer v/s benign).

Analytical Validation

Analytical validation established the performance characteristics of the test with standard analyte (SKBR3 cells), spiked into healthy donor blood to generate various dilutions (cell densities). These dilutions were processed as per the described procedures (proprietary differentially cytotoxic medium treatment and ICC profiling) to determine the yield of spiked cells. A summary of findings of the analytical validations is provided in Table 1.

Stability and Recovery

To determine the Analyte Stability, 27 × 5 mL aliquots of healthy donor blood were spiked with ~15 SKBR3 cells each (final = 3 cells / mL) and stored at 2°C - 8°C. Of the 27 aliquots, 9 aliquots each were used immediately or after 24h and 48h. Of the 9 aliquots evaluated at each time point, 3 aliquots were used to determine recovery of each cell type (multiplexed marker combinations). Additionally, 3 × 5 mL blood was collected from 5 known CTC+ cases of BrAD; one sample was processed immediately (0h), one after 24h at 2°C - 8°C and the third after 48h at 2°C - 8°C. Recoveries at 0h were normalized as 100% and recoveries at 24h and 48h were determined relative to the 0h recovery.

Finding: In the spiked samples, the recovery of EpCAM+ cells was 100.0%, 97.8% and 97.8% at 0h, 24h and 48h storage at 2°C - 8°C. Similarly, the recovery of GATA3+ cells was 100.0%, 93.3% and 91.1% at 0h, 24h and 48h, and that of GCDFP15+ cells was 100.0%, 100.0% and 97.8% at 0h, 24h and 48h (Supplementary Table S11). In clinical samples, the overall (combined PanCK+) recovery was 91.5% and 88.6% after 24h and 48h storage at 2°C - 8°C respectively when 0h recovery was normalized as

100% (Supplementary Table S12). The findings of the stability and recovery study indicated that the samples could be stored at 2°C-8°C for up to 48h with <15% loss of cells.

Linearity

SKBR3 cells were spiked into 264 × 5 mL aliquots of healthy donor blood samples, stored for 48h at 2°C - 8°C and then processed for recovery. The 264 aliquots comprised 3 sets of 88 aliquots (11 spikes × 8 replicates). The study also included 24 × 5 mL aliquots (3 sets × 8 replicates) of healthy donor blood samples which were not spiked. Each set was assigned to either of the 3 multiplexed marker combinations. Samples were stored for 48h at 2°C - 8°C. Linearity was evaluated by Linear Regression.

<u>Finding</u>: Recoveries of spiked cells were generally higher at spike densities of 5 cells / 5 mL and higher (Supplementary Figure S10). Coefficient of Determination (R^2) ≥0.98 in all markers indicated a significant linear response, especially in the range of 5 - 1280 cells / 5 mL.

Limits of Detection, Quantitation and Blank

The Limit of Blank (LoB) was determined from the 24 × 5 mL unspiked healthy female donor blood samples in the Linearity study. The Limit of Detection (LoD) was determined from a subset of the Linearity Study which included 72 × 5 mL samples spiked with 1, 3 or 5 SKBR3 cells (24 each). The Limit of Quantitation (LoQ) was determined from a subset of the Linearity Study which included 96 × 5 mL samples spiked with 1, 3, 5 or 10 SKBR3 cells (24 each).

Finding: No GATA3+, GCDFP15+ or EpCAM+ cells were detected in the unspiked samples, i.e., no false positives. Thus, the limit of blank (LoB) was determined to be 0 cells / mL. The limit of detection (LoD) was 1 cell / 5 mL. The Allowable Deviation from Linearity (ADL) was pre-specified at 15%. The LoQ was determined to be 10 cells / 5 mL for GATA3, GCDFP15 and EpCAM at which the deviations (%) from linearity were -5% for GATA3, -9% for GCDFP15 and -13% for EpCAM, all of which were within the permissible range of -26% to +22% for 15% ADL [3].

At the detection threshold of 15 cells / 5 mL for sample positivity, the observed deviation from linearity was between -5% (at 10 cells / 5 mL) and -7% (at 20 cells / 5 mL) for GATA3, between -9% (at 10 cells / 5 mL) and -14% (at 20 cells / 5 mL) for GCDFP15, and between -13% (at 10 cells / 5 mL) and -9% (at 20 cells / 5 mL) for EpCAM all of which were within the permissible range of -26% to +22% for 15% ADL. A second detection threshold was defined at 12 cells / 5 mL based on the ~15% reduction in recovery at 48 h observed in the analyte stability studies. The purpose of this second detection threshold is to identify those cases which may be incorrectly assigned as negative due to lower cell counts on account of losses during storage and transport at 2°C – 8°C. Samples with 12 – 15 CTCs / mL are hence termed as equivocal. For the purpose of the test, equivocal samples are considered positive. However, the performance metrics have also been derived with equivocal samples considered as negative to mitigate any confounding biases (**Supplementary Tables S5 and S7**).

Sensitivity, Specificity and Accuracy

SKBR3 cells were spiked into 50 × 15 mL aliquots (5 spikes × 10 replicates) of healthy donor blood at 15, 30, 60, 120 and 240 cells. Each 15 mL sample was split into 3 × 5 mL aliquots that were used for the analysis of each of the 3 marker combinations. Samples were stored for 48h at 2°C - 8°C prior to analysis. Unspiked healthy donor blood samples (30 × 5 mL) were included in this study for the determination of specificity. Samples with equivocal findings were considered as negative. Accuracy was determined based on total true positive and true negative samples detected out of the total 80 samples.

Finding: Among the 50 spiked samples evaluated for sensitivity, SKBR3 cells were detected in 47 samples, yielding a sensitivity of 94%. Since SKBR3 cells were undetectable in any of the 30 unspiked samples, the specificity was deemed to be 100%. (Supplementary Table S13). Accuracy, determined as the combined proportion of true positives and true negatives, was 96.3%.

Precision

On Day 1, User 1 spiked 15 (Low) SKBR3 cells into each of 8 × 5 mL aliquots of healthy donor blood, and 150 (High) SKBR3 cells into each of another 8 × 5 mL aliquots of healthy donor blood. All samples (8 Low spike + 8 High) were stored for 48h at 2°C - 8°C and processed. User 1 performed this study on 10 consecutive days and used one of two HCS Instruments. User 2 independently replicated the study on 10 consecutive days and used the second HCS Instrument. Mean Recoveries (%) were used to calculate Standard Deviation (SD) and Coefficient of Variation (CV, %) for Intra-Run, Inter-Run and Inter-Operator.

Finding: Precision of the test was determined across 2 operators in samples with high and low spike densities. Supplementary Table S14 provides the Coefficient of

Variation (CV, %) for intra-run, inter-run and inter-operator precision for low and high spike as well as the cumulative for all markers. The cumulative CV was ≤4% for intra-run, ≤0.5% for inter-operator and ≤1.9% for inter-run precision. The overall CV was 3.8% indicating high precision.

Robustness

Guard-band studies were performed to ascertain and establish the robustness of the assay by varying operating parameters within predefined limits. Criteria evaluated include incubation temperatures, incubation times, centrifugation speeds, buffer volumes, and antibody dilutions (Supplementary Table S15). Around 15 SKBR3 cells were spiked into 5 mL healthy donor blood samples which were processed as per the Test procedure. Each Guard-band parameter was evaluated with 9 samples, where 3 samples each were evaluated at (a) the normal range, (b) the higher range and (c) the lower range. Each parameter passed the Guard-band test if the variance was <10%.
Finding: The overall variability ranged from 0.9% - 5.9% indicating that controlled changes to test parameters do not adversely impact the performance. During routine test conditions, these variations are controlled via the use of standard operating procedures (SOP) and instrument calibration.

Interfering Substances

The performance characteristics of the Test were evaluated in presence of endogenous (pathology markers) and exogenous factors (non-anticancer drugs) as potential interfering agents (Supplementary Table S16). Pure (analytical grade) molecules for each of these agents were obtained from commercial vendors and stored under recommended conditions until use. All substances were reconstituted as

per manufacturer's instructions in appropriate solvents to prepare working stock solutions which were immediately used for spiking studies. All exogenous substances (drugs) were used at the reported medically relevant Peak Plasma Concentrations (C_{Max}) as per previously published literature, while endogenous substances (serum parameters) were evaluated at concentrations that are considered clinically elevated. Blood from a healthy donor (75 mL) who was not under any medication (last 14 days) was procured from a blood bank and spiked with about 750 SKBR3 cells. The spiked sample was split into 25 × 3 mL aliquots; 21 aliquots were spiked with each of the above substances at the indicated concentrations and 4 aliquots were used as unspiked controls. Each 3 mL sample was split into 3 × 1 mL aliquots; one aliquot each was used for detection of PanCK+, EpCAM+ cells; PanCK+, GATA3+ cells and PanCK+, GCDFP15+ cells respectively.

Finding: The presence of drugs at medically relevant peak plasma concentrations (C_{Max}) or the deranged serum parameters did not impact the recovery or detection of SKBR3 cells spiked into blood samples.

Phenotypic Characteristics of CTCs Detected in Clinical Validation Samples

The case control study employed an iterative cross validation design as explained in the Methods section. Details on the types of CTCs observed in various samples and their classification as negative / equivocal are provided below.

Among the samples from the 9,632 asymptomatic individuals in the case control study, cells positive for GATA3+(PanCK+, CD45-), GCDFP15+(PanCK+, CD45-) and EpCAM+(PanCK+, CD45-) were undetectable in all samples. Among samples from the 548 cancer cases in the case control study, there were 496 positives, 13 equivocals and 39 negatives. All 13 equivocal samples were positive for GCDFP15+

and EpCAM+ cells with undetectable GATA3+ cells (total greater than 12 cells). In 27 negative samples, GATA3+, GCDFP15+ and EpCAM+ cells were all undetectable. In 11 negative samples, GCDFP15+ and EpCAM+ cells were detected but GATA3+ cells were undetectable (total less than 12 cells). In 1 negative sample, GATA3+ and EpCAM+ cells were detected but GCDFP15+ cells were undetectable (total less than 12 cells).

Among the 112 cancer samples in the prospective study, there were 106 positives and 6 negatives. Among the negative samples, 4 were positive for GCDFP15+ cells and EpCAM+ cells with undetectable GATA3+ cells (total less than 12 cells) and in the remaining 2, GATA3+, GCDFP15+ and EpCAM+ cells were all undetectable. Among the 29 benign samples in the prospective study, there were 2 equivocals and 27 negatives. Among the 2 equivocal samples, one was positive for GATA3+ cells and GCDFP15+ cells with undetectable EpCAM+ cells (total greater than 12 cells) while the other was positive for GATA3+ cells and EpCAM+ cells with undetectable GCDFP15+ cells (total greater than 12 cells). The 27 negative samples included 24 where GATA3+, GCDFP15+ and EpCAM+ cells were all undetectable and 3 where GCDFP15+ cells were detectable but GATA3+ and EpCAM+ cells were undetectable.

REFERENCES

1. Crook, T.; Gaya, A.; Page, R.; Limaye, S.; Ranade, A.; Bhatt, A.; Patil, S.; Kumar, P.; Patil, D.; Akolkar, D. Clinical utility of circulating tumor-associated cells to predict and monitor chemo-response in solid tumors. *Cancer Chemother. Pharmacol.* **2021**, *87*, 197–205, doi:10.1007/s00280-020-04189-8.

2. Gaya, A.; Crook, T.; Plowman, N.; Ranade, A.; Limaye, S.; Bhatt, A.; Page, R.; Patil, R.; Fulmali, P.; Datta, V.; et al. Evaluation of circulating tumor cell clusters for pan-cancer noninvasive diagnostic triaging. *Cancer Cytopathol.* **2021**, *129*, 226–238, doi:10.1002/cncy.22366.

3. CLSI *Evaluation of Linearity of Quantitative Measurement Procedures - Table 3. Deviations from True Ratios for Different Percentages of ADL*; EP06Ed2.; 2020; ISBN 978-1-68440-096-6.

SUPPLEMENTARY FIGURES

Supplementary Figure S1. Design of Clinical Studies. The schema in (A) depicts the design of the Case Control Study as well as the Prospective Study. The schema in (B) depicts the correlation between Clinical Status and Prediction (by the test based on BrAD CTC detection) to determine the True Positives (TP), True Negatives (TN), False Positives (FP) and False Negatives (FN), which were used to determine the Sensitivity, Specificity and Accuracy of the test by standard definitions and formulae.

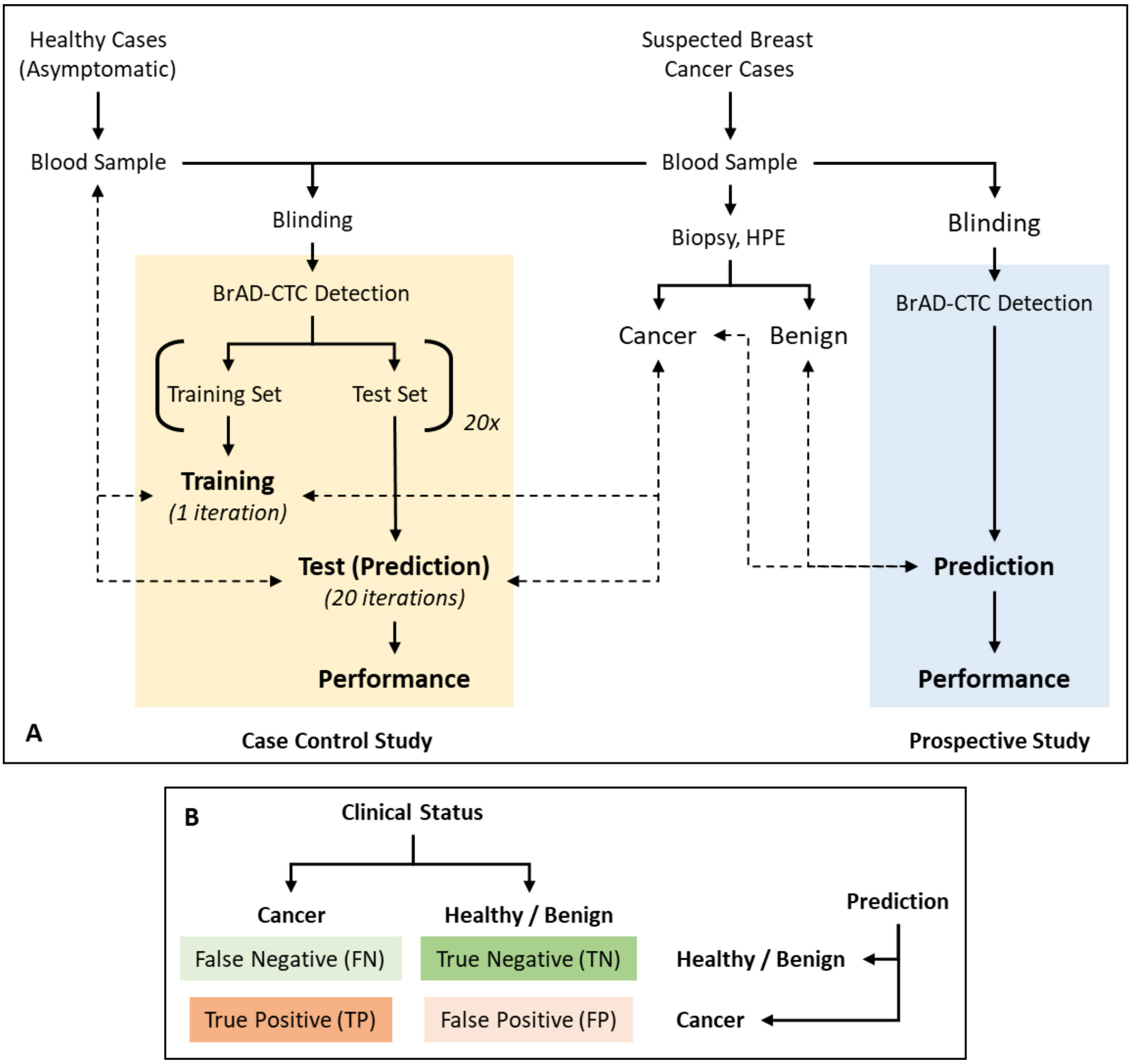

Supplementary Figure S2. Detection Thresholds.

SKBR3 (breast cancer) cells, MOLT3 (leukemia) cells, SW982 (sarcoma) cells, BrAD-CTCs, malignant breast tumor derived cells (M-TDCs) and benign breast tumor derived cells (B-TDCs) were immunostained to determine the expression level (FI: fluorescence intensity) of each marker.

A. GATA3

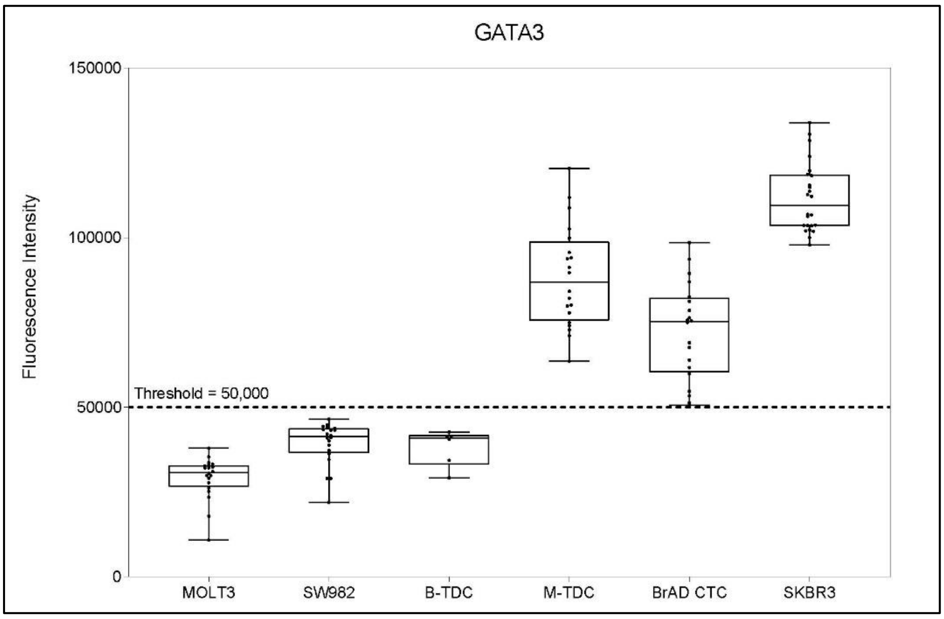

B. GCDFP15

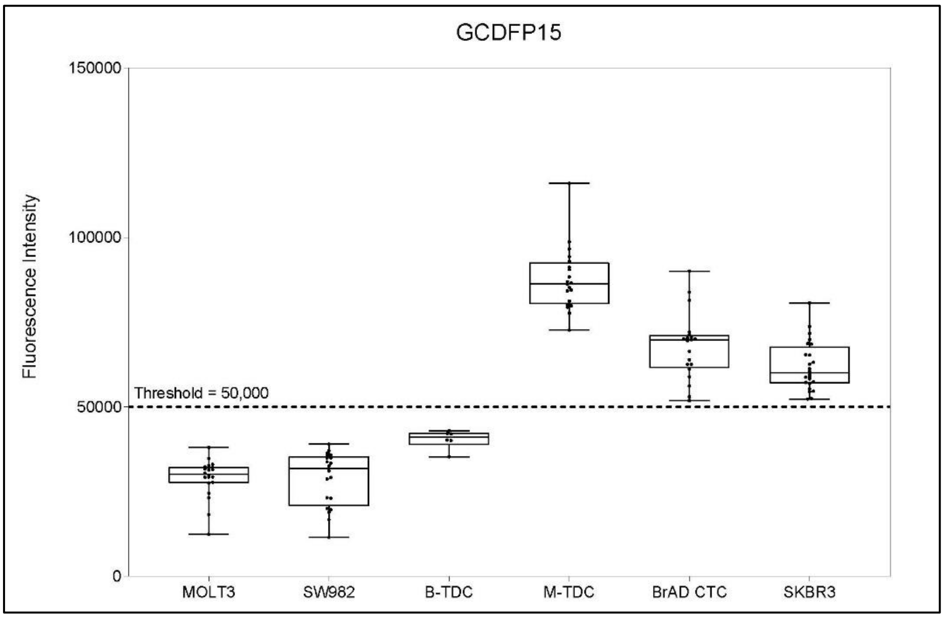

C. EpCAM

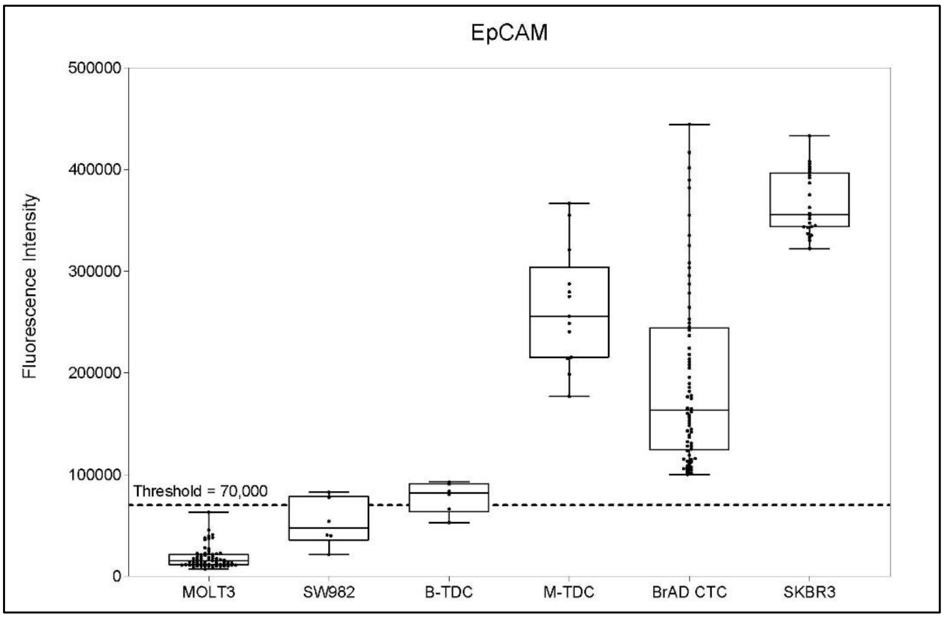

D. PanCK

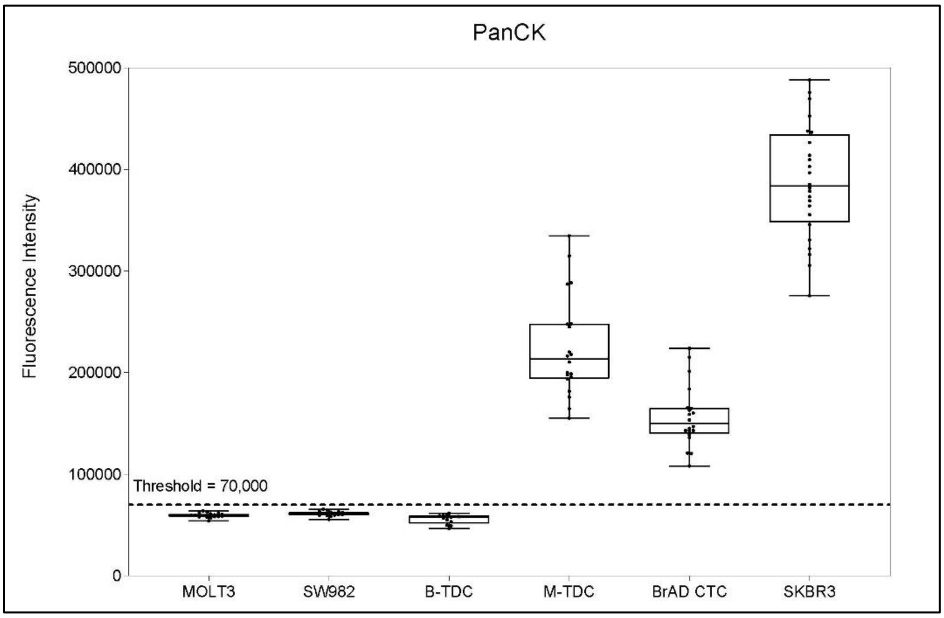

E. CD45

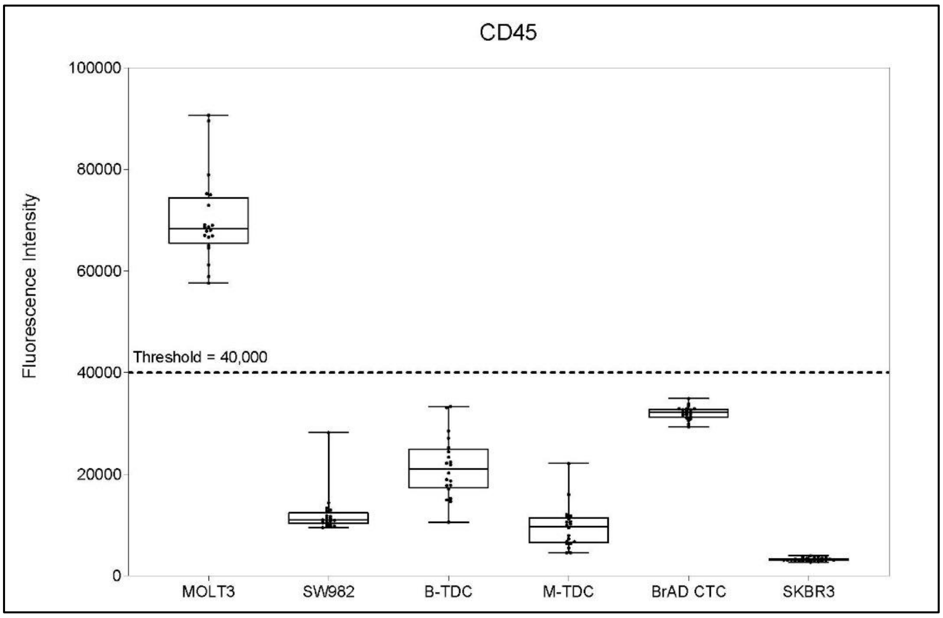

CD45
Fluorescence Intensity
100000
80000
60000
40000
20000
0
Threshold = 40,000
MOLT3
SW982
B-TDC
M-TDC
BrAD CTC
SKBR3

Supplementary Figure S3. Expression of GATA3 (A) and GCDFP15 (B) in various CTCs.

A. GATA3 in various CTCs

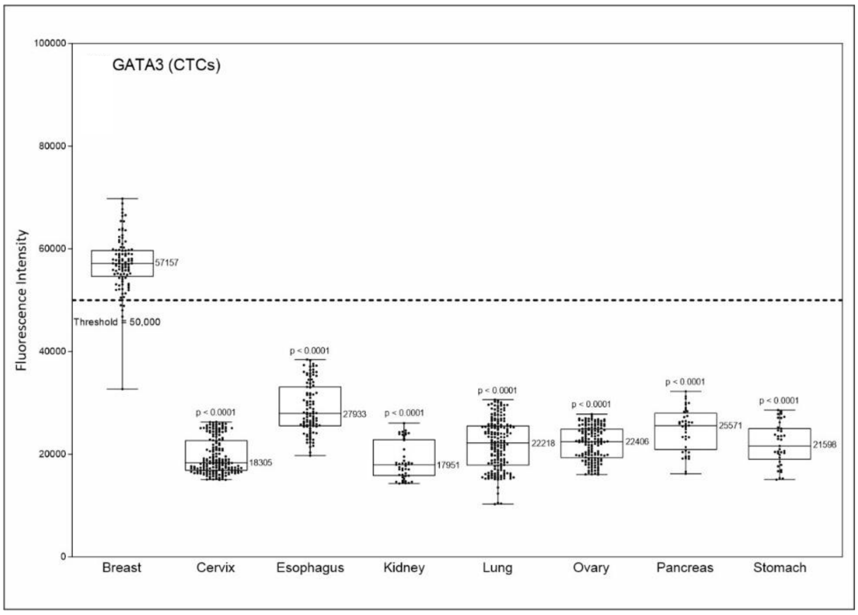

B. GCDFP15 in various CTCs

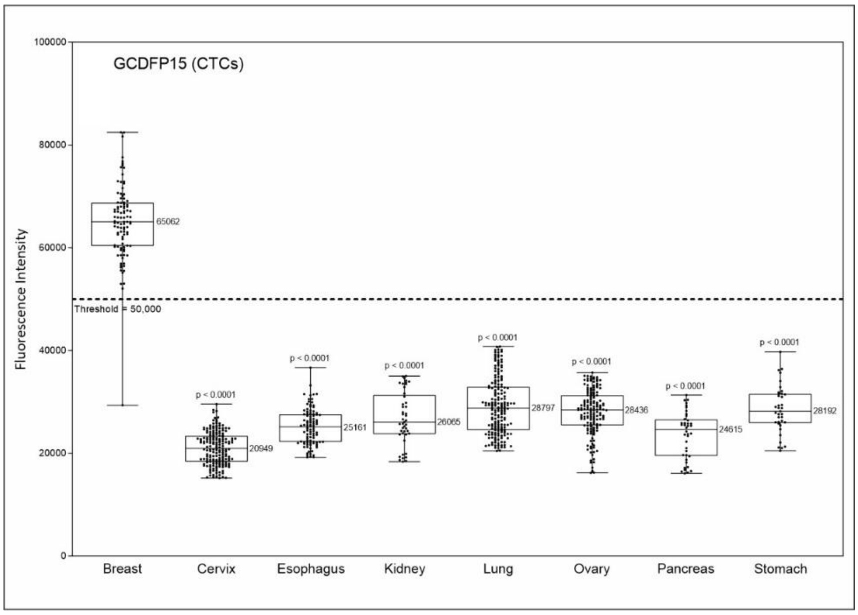

Supplementary Figure S4. Age-group and Marker Expression on CTCs. (A) GATA3, (B) GCDFP15, (C) EpCAM, (D) PanCK, (E) CD45

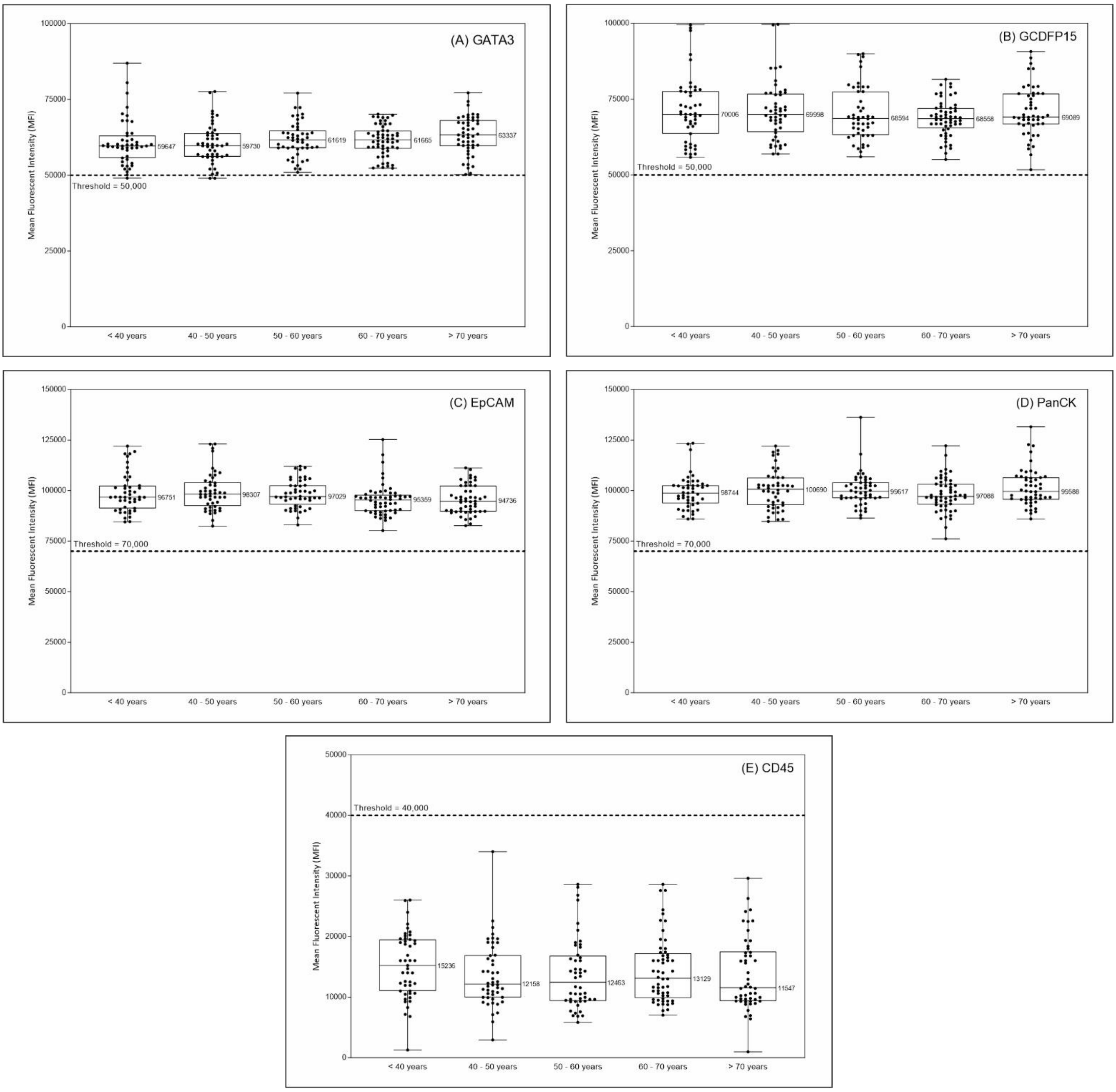

Supplementary Figure S5. Marker Expression on CTCs from Ductal and Lobular Subtypes.

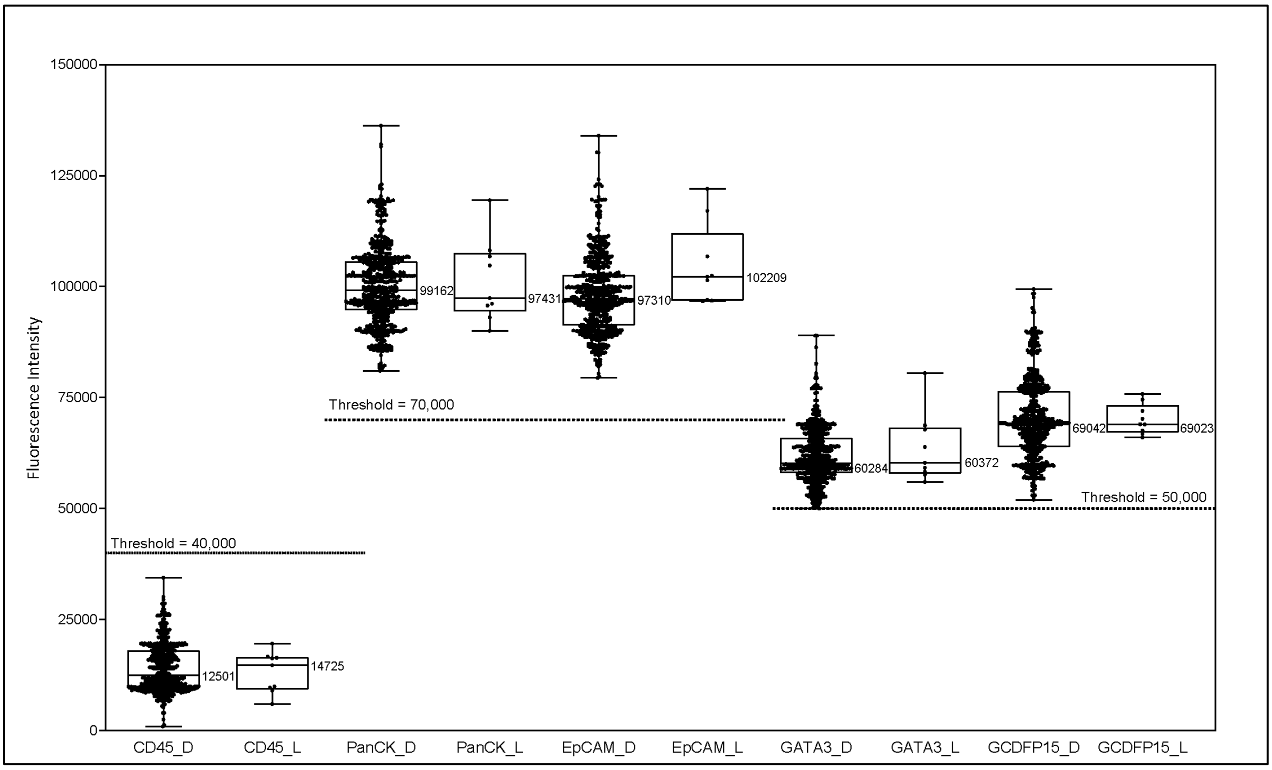

Supplementary Figure S6. Grade and Marker Expression on CTCs. (A) GATA3, (B) GCDFP15, (C) EpCAM, (D) PanCK, (E) CD45.

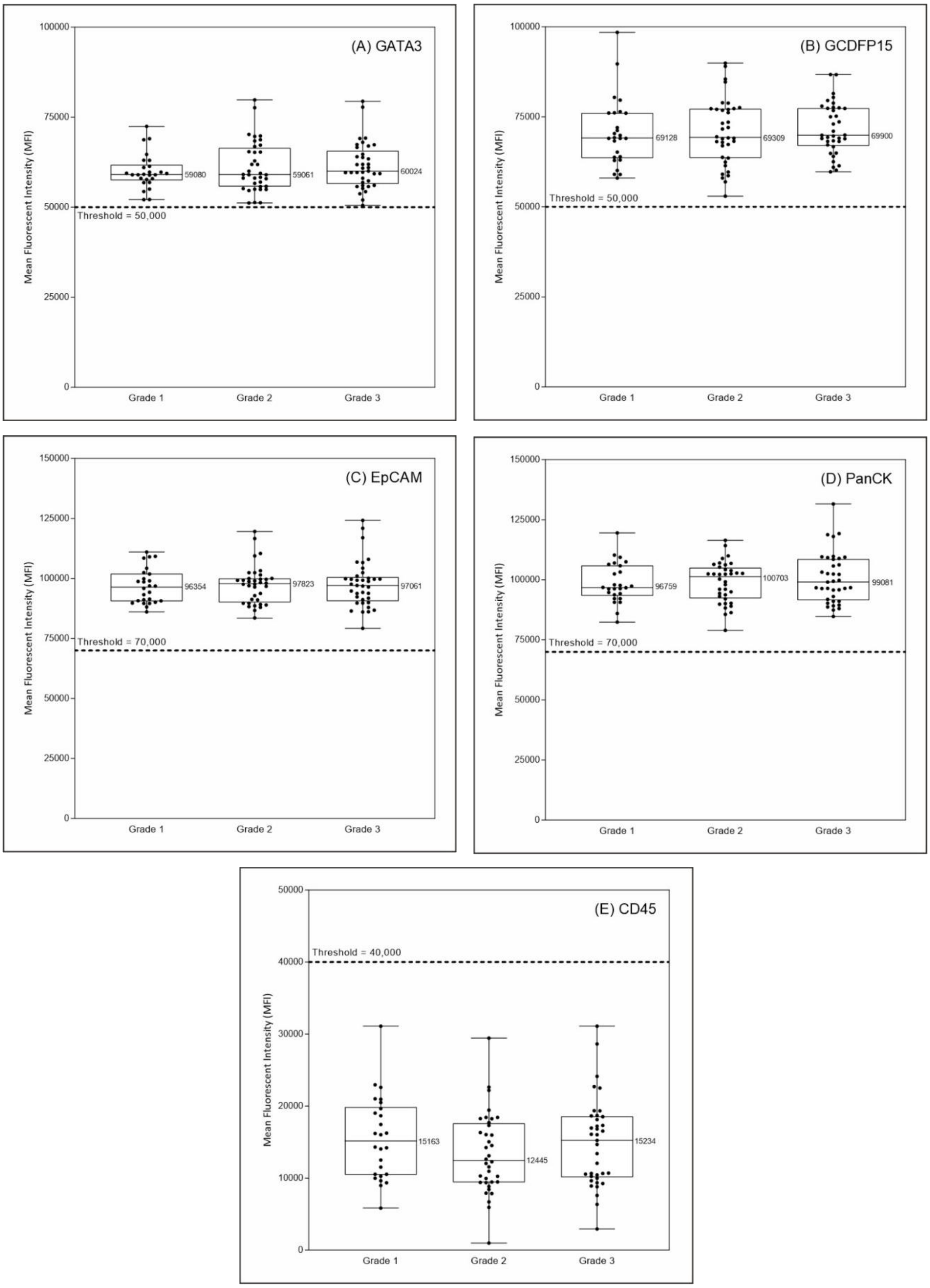

Supplementary Figure S7. Hormone Receptor Status and Marker Expression on CTCs. (A) GATA3, (B) GCDFP15, (C) EpCAM, (D) PanCK, (E) CD45.

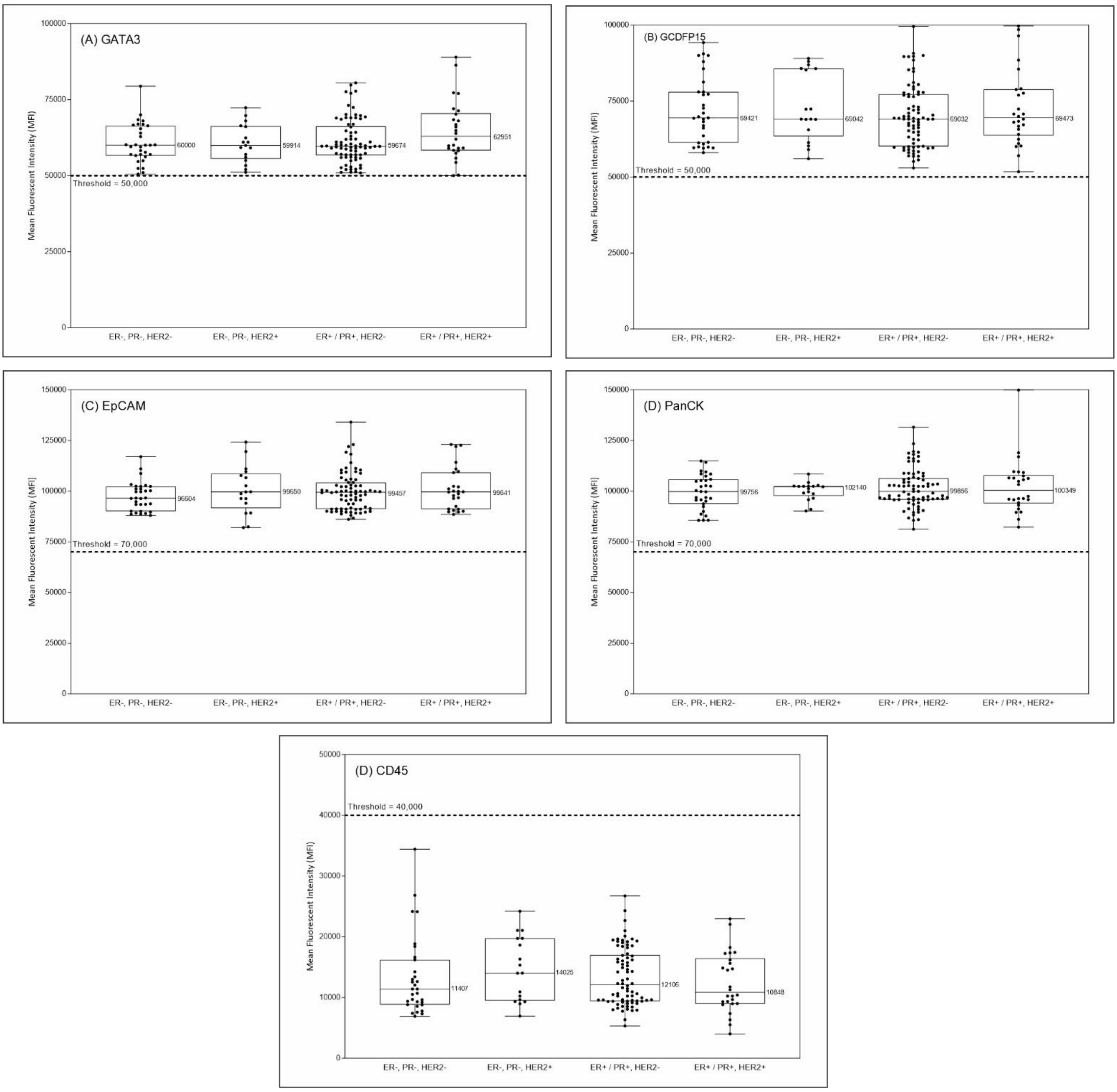

Supplementary Figure S8. Stage and Marker Expression on CTCs. (A) GATA3, (B) GCDFP15, (C) EpCAM, (D) PanCK, (E) CD45.

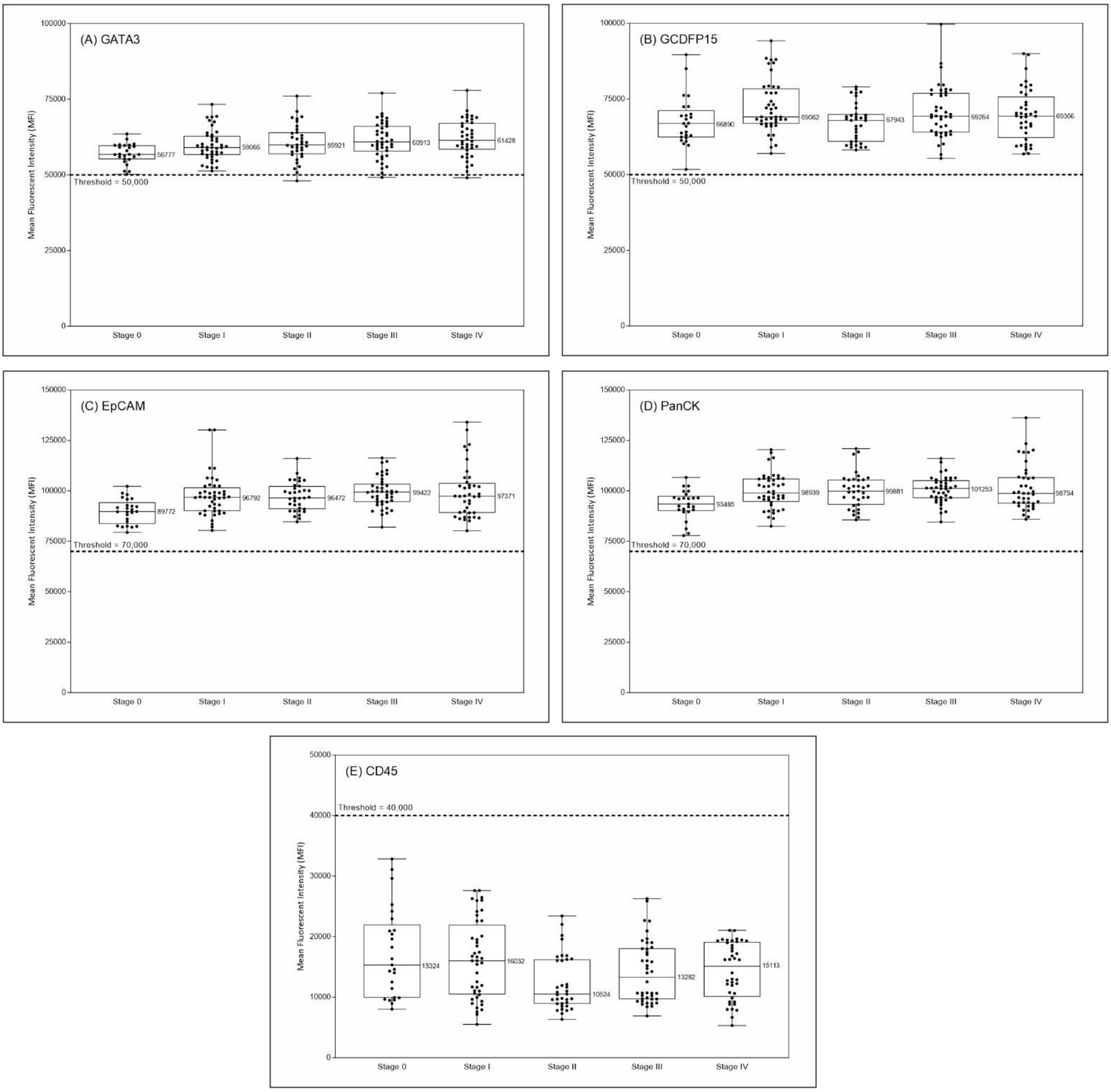

Supplementary Figure S9. Ethnicity and Marker Expression on CTCs.

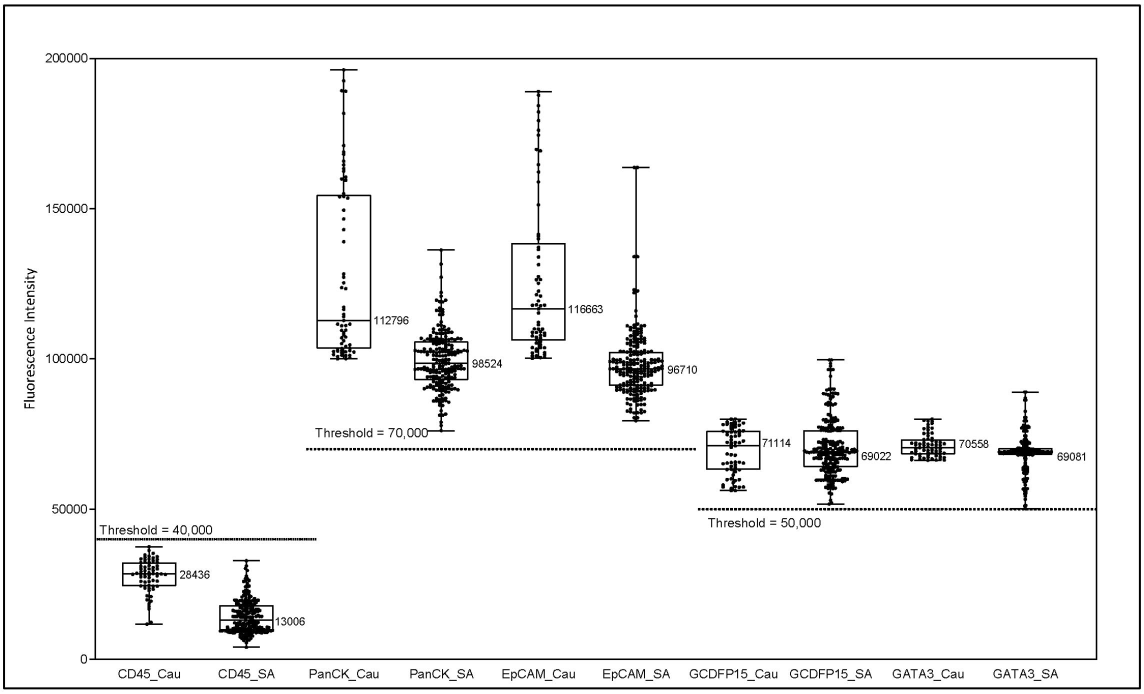

Supplementary Figure S10: Analytical Validation: Linearity. The Test exhibited significant linearity with R^2 ≥0.98. The Tabulated values below the figure show the recovery and range of recovery.

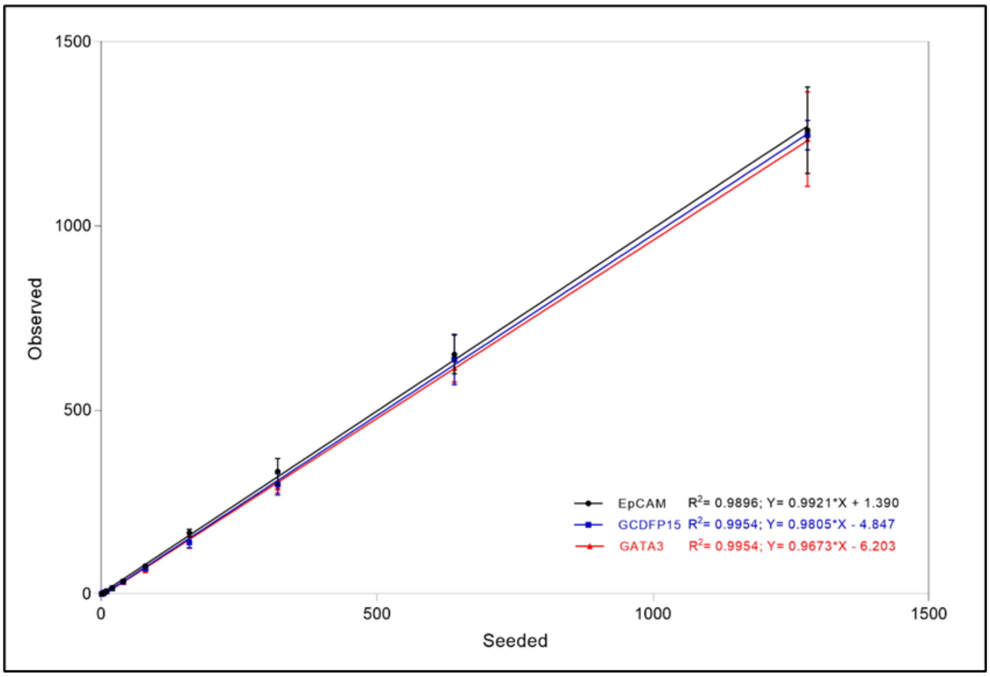

Spiked cells	Mean and % of Detected Cell Counts (8 Replicates)		
	PanCK+, EpCAM+	PanCK+, GATA3+	PanCK+, GCDFP15+
1280	1260.3 (98.5%)<br>1125 - 1380	1235.8 (96.5%)<br>1090 – 1473	1247.1 (97.4%)<br>1166 – 1291
640	651.0 (101.7%)<br>568 – 730	614.4 (96.0%)<br>561 – 689	637.3 (99.6%)<br>531 – 709
320	333.3 (104.1%)<br>285 – 385	290.1 (90.7%)<br>262 – 310	298.8 (93.4%)<br>250 – 335
160	165.8 (103.6%)<br>158 – 187	141.1 (88.2%)<br>120 – 168	140.6 (87.9%)<br>128 – 164
80	76.5 (95.6%)<br>69 – 85	64.6 (80.8%)<br>60 – 70	68.5 (85.6%)<br>62 – 72
40	34.8 (86.9%)<br>29 – 42	32.5 (81.3%)<br>29 – 34	33.6 (84.1%)<br>30 – 39
20	17.0 (85.0%)<br>16 – 19	16.0 (80.0%)<br>14 – 17	16.0 (80.0%)<br>14 – 20
10	8.1 (81.3%)<br>7 – 10	8.0 (80.0%)<br>7 – 9	8.3 (82.5%)<br>7 – 9
5	3.6 (72.5%)<br>3 – 4	3.5 (80.0%)<br>3 – 4	4.3 (85.0%)<br>3 - 6
3	2.9 (95.8%)<br>2 – 4	1.6 (54.2%)<br>1 – 2	2.0 (66.7%)<br>1 – 3
1	1.0 (100.0%)<br>1 - 1	0.4 (37.5%)<br>0 - 1	0.3 (25.0%)<br>0 - 1
0	0	0	0

SUPPLEMENTARY TABLES

Supplementary Table S1. Demographics of Case Control Validation Cohort.

	Cancer	Asymptomatic
**N = 10180**	**548**	**9,632**
**Age (years)**		
Median	52	50
Range	21 - 85	40 - 75
**Cancer Stage**		
0	32	-
I	157	
II	158	
III	100	
IV	101	

Supplementary Table S2. Eligibility Criteria for Case Control Study

	Cancer	Asymptomatic
**Inclusion**	• Adult females,<br>• Informed consent available,<br>• Willing for blood draw,<br>• No prior diagnosis of any other cancer,<br>• Recently diagnosed, therapy naïve cases,<br>• HPE diagnosis available,<br>• Stage data available,	• Adult females,<br>• Informed consent available,<br>• Willing for blood draw,<br>• No prior diagnosis of any cancer,<br>• No current suspicion of any cancer,<br>• BIRADS-I on Mammography
**Exclusion**	• Inability to provide informed consent or blood sample,<br>• Metachronous / synchronous malignancies,<br>• Pre-treated at the time of blood collection	• Inability to provide informed consent or blood sample,<br>• Diagnosed case of cancer,<br>• Suspicion of malignancy,<br>• BIRADS ≥ 2 or inconclusive / incomplete,

Supplementary Table S3. Demographics of Prospective Validation Cohort.

	Total Cases (n = 141)	*Of the total 141 cases:*	
		Cancer (n = 112)	***Benign (n = 29)***
**Age (years)**			
Median		*52*	*39*
Range	18 – 81	*21 - 81*	*18 - 69*
**Cancer Stage**			
0	-	*24*	-
I		*24*	
II		*24*	
III		*20*	
IV		*20*	
**Benign conditions**			
Benign fibrocystic disease	-	-	*2*
Benign parenchyma			*8*
Chronic inflammation			*1*
Fibroadenoma			*13*
Focal duct ectasia			*1*
Intraductal papilloma			*1*
Reactive lymphoid hyperplasia			*1*
Sclerosing adenosis			*1*
Focal intraductal hyperplasia			*1*

Supplementary Table S4. Eligibility Criteria for Prospective Study

Inclusion	Exclusion
• Adult females,<br>• Suspicion of breast cancer,<br>• No prior diagnosis of any other cancer,<br>• Informed consent available,<br>• Willing for blood draw,<br>• Willing to undergo standard diagnostic work up including biopsy for breast cancer,	• Inability to provide informed consent<br>• Inability to provide blood sample,<br>• Inability to undergo standard diagnostic work up including biopsy for breast cancer,<br>• Suspicion of any other type of malignancy,<br>• Previously diagnosed with any cancer,<br>• Received prior anticancer treatments,<br>• Non-availability of HPE diagnosis or stage data (later),

Supplementary Table S5. Validation Set Analysis

Cancer and asymptomatic samples were randomized into Training and Test Sets in a 70%:30% ratio. Subsequently, all samples were shuffled and random 30% assigned to Test Set 2. The shuffling and 30% randomization was repeated to generate Test Sets 3 - 20. The table reports the findings of the Training and 20 iterations of the Test Sets.

Set	Sample Type	Samples	Negative (%)	Equivocal (%)	Positive (%)
**Training**	**Asymptomatic**	**6742**	**6742 (100%)**	**-**	**-**
	**Cancers**	**384**	**30 (7.8%)**	**10 (2.6%)**	**344 (89.6%)**
	Stage 0	*22*	*8 (36.4%)*	*-*	*14 (63.6%)*
	Stage I	*110*	*12 (10.9%)*	*4 (3.6%)*	*94 (85.5%)*
	Stage II	*111*	*9 (8.1%)*	*5 (4.5%)*	*97 (87.4%)*
	Stage III	*70*	*1 (1.4%)*	*1 (1.4%)*	*68 (97.1%)*
	Stage IV	*71*	*-*	*-*	*71 (100%)*
**Test 1**	**Asymptomatic**	**2890**	**2890 (100%)**	**-**	**-**
	**Cancers**	**164**	**9 (5.5%)**	**3 (1.8%)**	**152 (92.7%)**
	Stage 0	*10*	*2 (20.0%)*	*-*	*8 (80.0%)*
	Stage I	*47*	*6 (12.8%)*	*1 (2.1%)*	*40 (85.1%)*
	Stage II	*47*	*1 (2.1%)*	*2 (4.3%)*	*44 (93.6%)*
	Stage III	*30*	*-*	*-*	*30 (100%)*
	Stage IV	*30*	*-*	*-*	*30 (100%)*
**Test 2**	**Asymptomatic**	**2890**	**2890 (100%)**	**-**	**-**
	**Cancers**	**164**	**16 (9.8%)**	**5 (3.0%)**	**143 (87.2%)**
	Stage 0	*10*	*3 (30.0%)*	*0 (0.0%)*	*7 (70.0%)*
	Stage I	*47*	*7 (14.9%)*	*2 (4.3%)*	*38 (80.9%)*
	Stage II	*47*	*5 (10.6%)*	*3 (6.4%)*	*39 (83.0%)*
	Stage III	*30*	*1 (3.3%)*	*-*	*29 (96.7%)*
	Stage IV	*30*	*-*	*-*	*30 (100%)*
**Test 3**	**Asymptomatic**	**2890**	**2890 (100%)**	**-**	**-**
	**Cancers**	**164**	**11 (6.7%)**	**9 (5.5%)**	**144 (87.8%)**
	Stage 0	*10*	*3 (30.0%)*	*-*	*7 (70.0%)*
	Stage I	*47*	*5 (10.6%)*	*5 (10.6%)*	*37 (78.7%)*
	Stage II	*47*	*2 (4.3%)*	*4 (8.5%)*	*41 (87.2%)*
	Stage III	*30*	*1 (3.3%)*	*-*	*29 (96.7%)*
	Stage IV	*30*	*-*	*-*	*30 (100%)*
**Test 4**	**Asymptomatic**	**2890**	**2890 (100%)**	**-**	**-**
	**Cancers**	**164**	**13 (7.9%)**	**3 (1.8%)**	**148 (90.2%)**
	Stage 0	*10*	*2 (20.0%)*	*-*	*8 (80.0%)*
	Stage I	*47*	*9 (19.1%)*	*2 (4.3%)*	*36 (76.6%)*
	Stage II	*47*	*2 (4.3%)*	*1 (2.1%)*	*44 (93.6%)*
	Stage III	*30*	*-*	*-*	*30 (100%)*
	Stage IV	*30*	*-*	*-*	*30 (100%)*
**Test 5**	**Asymptomatic**	**2890**	**2890 (100%)**	**-**	**-**
	**Cancers**	**164**	**11 (6.7%)**	**3 (1.8%)**	**150 (91.5%)**
	Stage 0	*10*	*3 (30.0%)*	*-*	*7 (70.0%)*
	Stage I	*47*	*6 (12.8%)*	*1 (2.1%)*	*40 (85.1%)*
	Stage II	*47*	*2 (4.3%)*	*2 (4.3%)*	*43 (91.5%)*
	Stage III	*30*	*-*	*-*	*30 (100%)*
	Stage IV	*30*	*-*	*-*	*30 (100%)*
**Test 6**	**Asymptomatic**	**2890**	**2890 (100%)**	**-**	**-**

Set	Sample Type	Samples	Negative (%)	Equivocal (%)	Positive (%)
	**Cancers**	**164**	**9 (5.5%)**	**5 (3.0%)**	**150 (91.5%)**
	Stage 0	*10*	*3 (30.0%)*	*-*	*7 (70.0%)*
	Stage I	*47*	*4 (8.5%)*	*4 (8.5%)*	*39 (83.0%)*
	Stage II	*47*	*2 (4.3%)*	*1 (2.1%)*	*44 (93.6%)*
	Stage III	*30*	*-*	*-*	*30 (100%)*
	Stage IV	*30*	*-*	*-*	*30 (100%)*
**Test 7**	**Asymptomatic**	**2890**	**2890 (100%)**	**-**	**-**
	**Cancers**	**164**	**14 (8.5%)**	**4 (2.4%)**	**146 (89.0%)**
	Stage 0	*10*	*4 (40.0%)*	*-*	*6 (60.0%)*
	Stage I	*47*	*6 (12.8%)*	*1 (2.1%)*	*40 (85.1%)*
	Stage II	*47*	*4 (8.5%)*	*3 (6.4%)*	*40 (85.1%)*
	Stage III	*30*	*-*	*-*	*30 (100%)*
	Stage IV	*30*	*-*	*-*	*30 (100%)*
**Test 8**	**Asymptomatic**	**2890**	**2890 (100%)**	**-**	**-**
	**Cancers**	**164**	**11 (6.7%)**	**3 (1.8%)**	**150 (91.5%)**
	Stage 0	*10*	*1 (10.0%)*	*-*	*9 (90.0%)*
	Stage I	*47*	*5 (10.6%)*	*-*	*42 (89.4%)*
	Stage II	*47*	*4 (8.5%)*	*2 (4.3%)*	*41 (87.2%)*
	Stage III	*30*	*1 (3.3%)*	*1 (3.3%)*	*28 (93.3%)*
	Stage IV	*30*	*-*	*-*	*30 (100%)*
**Test 9**	**Asymptomatic**	**2890**	**2890 (100%)**	**-**	**-**
	**Cancers**	**164**	**15 (9.1%)**	**1 (0.6%)**	**148 (90.2%)**
	Stage 0	*10*	*5 (50.0%)*	*-*	*5 (50.0%)*
	Stage I	*47*	*7 (14.9%)*	*1 (2.1%)*	*39 (83.0%)*
	Stage II	*47*	*2 (4.3%)*	*-*	*45 (95.7%)*
	Stage III	*30*	*1 (3.3%)*	*-*	*29 (96.7%)*
	Stage IV	*30*	*-*	*-*	*30 (100%)*
**Test 10**	**Asymptomatic**	**2890**	**2890 (100%)**	**-**	**-**
	**Cancers**	**164**	**8 (4.9%)**	**4 (2.4%)**	**152 (92.7%)**
	Stage 0	*10*	*2 (20.0%)*	*-*	*8 (80.0%)*
	Stage I	*47*	*5 (10.6%)*	*1 (2.1%)*	*41 (87.2%)*
	Stage II	*47*	*1 (2.1%)*	*3 (6.4%)*	*43 (91.5%)*
	Stage III	*30*	*-*	*-*	*30 (100%)*
	Stage IV	*30*	*-*	*-*	*30 (100%)*
**Test 11**	**Asymptomatic**	**2890**	**2890 (100%)**	**-**	**-**
	**Cancers**	**164**	**13 (7.9%)**	**5 (3.0%)**	**146 (89.0%)**
	Stage 0	*10*	*2 (20.0%)*	*-*	*8 (80.0%)*
	Stage I	*47*	*6 (12.8%)*	*2 (4.3%)*	*39 (83.0%)*
	Stage II	*47*	*5 (10.6%)*	*2 (4.3%)*	*40 (85.1%)*
	Stage III	*30*	*-*	*1 (3.3%)*	*29 (96.7%)*
	Stage IV	*30*	*-*	*-*	*30 (100%)*
**Test 12**	**Asymptomatic**	**2890**	**2890 (100%)**	**-**	**-**
	**Cancers**	**164**	**16 (9.8%)**	**3 (1.8%)**	**145 (88.4%)**
	Stage 0	*10*	*4 (40.0%)*	*-*	*6 (60.0%)*
	Stage I	*47*	*7 (14.9%)*	*1 (2.1%)*	*39 (83.0%)*
	Stage II	*47*	*4 (8.5%)*	*2 (4.3%)*	*41 (87.2%)*
	Stage III	*30*	*1 (3.3%)*	*-*	*29 (96.7%)*
	Stage IV	*30*	*-*	*-*	*30 (100%)*
**Test 13**	**Asymptomatic**	**2890**	**2890 (100%)**	**-**	**-**
	**Cancers**	**164**	**13 (7.9%)**	**4 (2.4%)**	**147 (89.6%)**
	Stage 0	*10*	*2 (20.0%)*	*-*	*8 (80.0%)*
	Stage I	*47*	*8 (17.0%)*	*1 (2.1%)*	*38 (80.9%)*
	Stage II	*47*	*3 (6.4%)*	*2 (4.3%)*	*42 (89.4%)*
	Stage III	*30*	*-*	*1 (3.3%)*	*29 (96.7%)*
	Stage IV	*30*	*-*	*-*	*30 (100%)*
**Test 14**	**Asymptomatic**	**2890**	**2890 (100%)**	**-**	**-**

Set	Sample Type	Samples	Negative (%)	Equivocal (%)	Positive (%)
	**Cancers**	**164**	**13 (7.9%)**	**2 (1.2%)**	**149 (90.9%)**
	Stage 0	*10*	*3 (30.0%)*	*-*	*7 (70.0%)*
	Stage I	*47*	*5 (10.6%)*	*-*	*42 (89.4%)*
	Stage II	*47*	*4 (8.5%)*	*2 (4.3%)*	*41 (87.2%)*
	Stage III	*30*	*1 (3.3%)*	*-*	*29 (96.7%)*
	Stage IV	*30*	*-*	*-*	*30 (100%)*
**Test 15**	**Asymptomatic**	**2890**	**2890 (100%)**	**-**	**-**
	**Cancers**	**164**	**15 (9.1%)**	**3 (1.8%)**	**146 (89.0%)**
	Stage 0	*10*	*5 (50.0%)*	*-*	*5 (50.0%)*
	Stage I	*47*	*5 (10.6%)*	*1 (2.1%)*	*41 (87.2%)*
	Stage II	*47*	*5 (10.6%)*	*1 (2.1%)*	*41 (87.2%)*
	Stage III	*30*	*-*	*1 (3.3%)*	*29 (96.7%)*
	Stage IV	*30*	*-*	*-*	*30 (100%)*
**Test 16**	**Asymptomatic**	**2890**	**2890 (100%)**	**-**	**-**
	**Cancers**	**164**	**11 (6.7%)**	**1 (0.6%)**	**152 (92.7%)**
	Stage 0	*10*	*4 (40.0%)*	*-*	*6 (60.0%)*
	Stage I	*47*	*4 (8.5%)*	*1 (2.1%)*	*42 (89.4%)*
	Stage II	*47*	*2 (4.3%)*	*-*	*45 (95.7%)*
	Stage III	*30*	*1 (3.3%)*	*-*	*29 (96.7%)*
	Stage IV	*30*	*-*	*-*	*30 (100%)*
**Test 17**	**Asymptomatic**	**2890**	**2890 (100%)**	**-**	**-**
	**Cancers**	**164**	**9 (5.5%)**	**4 (2.4%)**	**151 (92.1%)**
	Stage 0	*10*	*2 (20.0%)*	*-*	*8 (80.0%)*
	Stage I	*47*	*4 (8.5%)*	*1 (2.1%)*	*42 (89.4%)*
	Stage II	*47*	*3 (6.4%)*	*2 (4.3%)*	*42 (89.4%)*
	Stage III	*30*	*-*	*1 (3.3%)*	*29 (96.7%)*
	Stage IV	*30*	*-*	*-*	*30 (100%)*
**Test 18**	**Asymptomatic**	**2890**	**2890 (100%)**	**-**	**-**
	**Cancers**	**164**	**13 (7.9%)**	**2 (1.2%)**	**149 (90.9%)**
	Stage 0	*10*	*5 (50.0%)*	*-*	*5 (50.0%)*
	Stage I	*47*	*7 (14.9%)*	*1 (2.1%)*	*39 (83.0%)*
	Stage II	*47*	*1 (2.1%)*	*1 (2.1%)*	*45 (95.7%)*
	Stage III	*30*	*-*	*-*	*30 (100%)*
	Stage IV	*30*	*-*	*-*	*30 (100%)*
**Test 19**	**Asymptomatic**	**2890**	**2890 (100%)**	**-**	**-**
	**Cancers**	**164**	**8 (4.9%)**	**4 (2.4%)**	**152 (92.7%)**
	Stage 0	*10*	*2 (20.0%)*	*-*	*8 (80.0%)*
	Stage I	*47*	*5 (10.6%)*	*-*	*42 (89.4%)*
	Stage II	*47*	*1 (2.1%)*	*4 (8.5%)*	*42 (89.4%)*
	Stage III	*30*	*-*	*-*	*30 (100%)*
	Stage IV	*30*	*-*	*-*	*30 (100%)*
**Test 20**	**Asymptomatic**	**2890**	**2890 (100%)**	**-**	**-**
	**Cancers**	**164**	**13 (7.9%)**	**4 (2.4%)**	**147 89.6%)**
	Stage 0	*10*	*4 (40.0%)*	*-*	*6 (60.0%)*
	Stage I	*47*	*4 (8.5%)*	*2 (4.3%)*	*41 (87.2%)*
	Stage II	*47*	*4 (8.5%)*	*2 (4.3%)*	*41 (87.2%)*
	Stage III	*30*	*1 (3.3%)*	*-*	*29 (96.7%)*
	Stage IV	*30*	*-*	*-*	*30 (100%)*

Supplementary Table S6. Expanded findings of the Case Control Clinical Validation Study. The table below indicates the Median and range of Sensitivities as well as the corresponding Accuracies for cumulative and cancer stage-wise, as observed in the 20-fold cross-validation. 95% confidence interval (CI) are provided for the median values of sensitivity and accuracy. In one analysis for determination of sensitivity and accuracy, samples with equivocal findings were considered as negative and in the other analysis, samples with equivocal findings were considered as positive. Since the test recommends clinical follow-up in individuals with equivocal findings, the final reported values for sensitivity and accuracy (Table 2) are based on samples with equivocal findings being considered as positive. Since none of the samples in the control (cancer-free and asymptomatic) cohort had positive or equivocal findings, overall specificity was 100%.

	**Considering Equivocal Findings as Negative**		**Considering Equivocal Findings as Positive**	
	**Sensitivity**	**Accuracy**	**Sensitivity**	**Accuracy**
**Cumulative**	Median: 90.55%<br>Range: 87.20% - 92.68%<br>95%CI: 89.51% - 91.59%	Median: 99.49%<br>Range: 99.31% - 99.61%<br>95%CI: 99.24% - 99.74%	Median: 92.07%<br>Range: 90.24% - 99.58%<br>95%CI: 91.12% - 93.03%	Median: 99.57%<br>Range: 99.48% - 99.74%<br>95%CI: 99.34% - 99.81%
Stage 0	Median: 70.00%<br>Range: 50.00% - 99.98%<br>95%CI: 34.75% - 93.33%	Median: 99.90%<br>Range: 99.83% - 99.97%<br>95%CI: 99.70% - 99.98%	Median: 70.00%<br>Range: 50.00% - 99.98%<br>95%CI: 34.75% - 93.33%	Median: 99.90%<br>Range: 99.83% - 99.97%<br>95%CI: 99.70% - 99.98%
Stage I	Median: 85.11%	Median: 99.76%	Median: 89.36%	Median: 99.81%

	Range: 76.60% - 89.36%<br>95%CI: 71.69% - 93.80%	Range: 99.63% - 99.83%<br>95%CI: 99.51% - 99.90%	Range: 80.85% - 99.82%<br>95%CI: 76.90% - 96.45%	Range: 99.69% - 99.86%<br>95%CI: 99.60% - 99.94%
Stage II	Median: 89.36%<br>Range: 82.98% - 95.74%<br>95%CI: 76.90% - 96.45%	Median: 99.83%<br>Range: 99.73% - 99.93%<br>95%CI: 99.60% - 99.94%	Median: 95.74%<br>Range: 89.36% - 99.87%<br>95%CI: 85.46% - 99.48%	Median: 99.91%<br>Range: 99.83% - 99.97%<br>95%CI: 99.75% - 99.99%
Stage III	Median: 96.67%<br>Range: 93.33% - 100.0%<br>95%CI: 82.78% - 99.92%	Median: 99.97%<br>Range: 99.93% - 100.0%<br>95%CI: 99.81% - 100.00%	Median: 100.0%<br>Range: 96.67% - 100.0%<br>95%CI: 88.43% - 100.00%	Median: 100.0%<br>Range: 99.97% - 100.0%<br>95%CI: 99.87% - 100.00%
Stage IV	Median: 100.0%<br>Range: 100.0% - 100.0%<br>95%CI: 88.43% - 100.00%	Median: 100.0%<br>Range: 100.0% - 100.0%<br>95%CI: 99.87% - 100.00%	Median: 100.0%<br>Range: 100.0% - 100.0%<br>95%CI: 88.43% - 100.00%	Median: 100.0%<br>Range: 100.0% - 100.0%<br>95%CI: 99.87% - 100.00%

Supplementary Table S7. Prospective Validation Cohort findings

Sample Type	Samples	Negative (%)	Equivocal (%)	Positive (%)
**Benign**	**29**	**27 (93.1%)**	**2 (6.9%)**	**-**
**Cancers**	**112 (94.6%)**	**6 (5.4%)**	**-**	**106**
Stage 0	*24*	*3 (2.5%)*	-	*21 (87.5%)*
Stage I	*24*	*1 (4.2%)*	-	*23 (95.8%)*
Stage II	*24*	*1 (4.2%)*	-	*23 (95.8%)*
Stage III	*20*	*1 (5.0%)*	-	*19 (95.0%)*
Stage IV	*20*	-	-	*20 (100%)*

Supplementary Table S8. Expanded findings of the Prospective Clinical Validation Study. The table below indicates the stage-wise and cumulative (all stages) sensitivity and accuracy of the test. Among the 141 samples, there were 2 samples with equivocal test findings. Both samples were subsequently diagnosed with benign conditions of the breast. Considering both samples with equivocal findings as negative and as positive, the specificity of the test was 100% and 93.1% respectively (cancer v/s benign). Accuracy was determined based on the differential analysis of samples with equivocal findings.

	**Sensitivity**	**Accuracy**	
		**Equivocal Samples as Negative**	**Equivocal Samples as Positives**
**Cumulative**	94.64%<br>95% CI: 88.70% - 98.01%	95.74%<br>95% CI: 90.97% - 98.42%	94.33%<br>95% CI: 89.13% - 97.52%
Stage 0	87.50%<br>95% CI: 67.64% - 97.34%	94.34%<br>95% CI: 84.34% - 98.82%	90.57%<br>95% CI: 79.34% - 96.87%
Stage I	95.83%<br>95% CI: 78.88% - 99.89%	98.11%<br>95% CI: 89.93% - 99.95%	94.34%<br>95% CI: 84.34% - 98.82%
Stage II	95.83%<br>95% CI: 78.88% - 99.89%	98.11%<br>95% CI: 89.93% - 99.95%	94.34%<br>95% CI: 84.34% - 98.82%
Stage III	95.00%<br>95% CI: 75.13% - 99.87%	97.96%<br>95% CI: 89.15% - 99.95%	93.88%<br>95% CI: 83.13% - 98.72%
Stage IV	100.00%<br>95% CI: 83.16% - 100.00%	100.00%<br>95% CI: 92.75% - 100.00%	95.92%<br>95% CI: 86.02% - 99.50%

Supplementary Table S9. Findings of TaqMan ddPCR Assays. The table below indicates the various gene variants detected in the tissue samples of 53 breast cancer cases by NGS. ddPCR analysis of these variants in genomic DNA isolated from CTC enriched WBCs, showed an overall 81.1% concordance with findings on tumor tissue.

**Target Assay**	**Gene ID**	**Control Plasmids**	**Samples**		
			**Total**	**Positives**	**Negatives**
AKT1_E17K	AHWSLXQ	34R2	7	5	2
EGFR_D855N	AHFBBKR		2	2	0
ESR1_Y537N	AHCTE56		3	3	0
GNAS_R201C	AH6R7EO	24R3	1	1	0
GNAS_R201H	AH705KW		3	2	1
KRAS_G12C	AHHS7X9		1	1	0
PIK3CA_E545Q	AH21CV0		1	0	1
PIK3CA_E542K	AHKA4AQ	24R5	7	5	2
PIK3CA_E545K	AHLJ2GY		8	6	2
PIK3CA_H1047L	AHPAWZM	24R5	1	1	0
PIK3CA_H1047R	AHD2DCF		9	8	1
PIK3CA_N345K	AHHS7YA		2	2	0
TP53_R248Q	AHVJNUO	24R3	3	3	0
TP53_R248W	AHRSTB0		1	0	1
TP53_R249S	AHX1J64		1	1	0
TP53_R273H	AHUAPOG		2	2	0
KRAS_G12V	AH0JGKY	Internal	1	1	0
**OVERALL**			**53**	**43 (81.1%)**	**10**

Supplementary Table S10. Demographics of Cohort with Benign or Inflammatory Breast Conditions

Parameter	Value
**Median Age (Range)**	34 years (19 – 72 years)
**Diagnosis**	
Adenosis	2
Benign Breast Disease	13
Benign breast parenchyma	15
Benign Duct Papilloma	1
Benign Fibrocystic Disease	2
Benign Fibroepithelial lesion	1
Benign Phyllodes	2
Benign Proliferative Disease	2
Duct Ectasia	3
Fibroadenoma	29
Focal intraductal Hyperplasia	3
Intraductal papilloma	1
Lactating adenoma	1
Lipoma	1
Mastitis	13
Reactive lymphoid hyperplasia	1
Squamous metaplasia	1

Supplementary Table S11. Analytical Validation: Stability and Recovery of Spiked Cells. SKBR3 cells were spiked into healthy donor blood samples and the recovery of spiked cells was evaluated for up to 48 hours.

**Time (h)**	**Spiked Cells**	**Mean Recovery, % Recovery and Recovery Range (%)**		
		**PanCK+, EpCAM+**	**PanCK+, GATA3+**	**PanCK+, GCDFP15+**
0	15	15.0 (100%)<br>93.3% - 106.7%	15.0 (100%)<br>93.3% - 106.7%	15 (100%)<br>93.3% - 106.7%
24	15	14.7 (97.8%)<br>93.3% - 100.0%	14.0 (93.3%)<br>86.7% - 100.0%	15 (100%)<br>93.3% - 106.7%
48	15	14.7 (97.8%)<br>93.3% - 100.0%	13.7 (91.1%)<br>86.7% - 93.3%	14.7 (97.8%)<br>93.3% - 100.0%

Supplementary Table S12. Analytical Validation Stability and Recovery of CTCs in Clinical Samples. Blood samples from known BrAD-CTC positive cases were evaluated for recovery of BrAD CTCs for up to 48 hours.

Time (h)	Cell Types, Detected Numbers, % Recovery and % Recovery Range								
	**Patient**	**(a) PanCK+, EpCAM+**		**(b) PanCK+, GATA3+ cells**		**(c) PanCK+, GCDFP15+ cells**		**Total PanCK+ cells (a + b + c)**	
		**Cells**	**Recovery**	**Cells**	**Recovery**	**Cells**	**Recovery**	**Cells**	**Recovery**
**0 h**	P_1	6	***100%***	5	***100%***	6	***100%***	17	***100%***
	P_2	8		6		6		20	
	P_3	6		6		7		19	
	P_4	8		5		6		19	
	P_5	7		6		8		21	
**24 h**	P_1	5	83.3%	5	100.0%	5	83.3%	15	88.2%
	P_2	7	87.5%	5	83.3%	5	83.3%	17	85.0%
	P_3	6	100.0%	5	83.3%	6	85.7%	17	89.5%
	P_4	7	87.5%	5	100.0%	6	100.0%	18	94.7%
	P_5	7	100.0%	6	100.0%	8	100.0%	21	100.0%
	Mean (Range)	91.7% (83.3% - 100.0%)		93.3% (83.3% - 100.0%)		90.5% (83.3% - 100.0%)		91.5% (85.0% - 100.0%)	
**48 h**	P_1	5	83.3%	5	100.0%	5	83.3%	15	88.2%
	P_2	7	87.5%	5	83.3%	6	100.0%	18	90.0%
	P_3	5	83.3%	5	83.3%	6	85.7%	16	84.2%
	P_4	7	87.5%	6	120.0%	5	83.3%	18	94.7%
	P_5	6	85.7%	5	83.3%	7	87.5%	18	85.7%
	Mean (Range)	85.5% (83.3% - 100.0%)		94.0% (83.3% - 120.0%)		88.0% (83.3% - 100.0%)		88.6% (84.2% - 94.7%)	

Supplementary Table S13. Analytical Validation: Sensitivity, Specificity, Accuracy. SKBR3 cells were spiked into healthy donor blood samples at various seed densities and their recoveries evaluated to determine sensitivity. Unspiked healthy donor blood samples were evaluated for false positives to determine specificity. Accuracy was determined from sensitivity and specificity.

Spiked	Detected Cells: Mean (Range)	Negative	Positive
**PanCK+, EpCAM+, CD45-**			
**0**	-	30	-
**5***	3.9 (2 – 5)	2	8
**10***	9.6 (8 – 10)	-	10
**20***	18.8 (16 – 22)	-	10
**40***	41.4 (34 – 44)	-	10
**80***	76.1 (71 - 80)	-	10
**PanCK+, GATA3+, CD45-**			
**0**	-	30	-
**5***	4.4 (3 – 5)	1	9
**10***	9.2 (6 – 10)	-	10
**20***	19.7 (16 – 20)	-	10
**40***	40.4 (32 – 40)	-	10
**80***	74.3 (70 - 79)	-	10
**PanCK+, GCDFP15+, CD45-**			
**0**	-	30	-
**5***	2.9 (2 – 4)	3	7
**10***	6.9 (6 – 7)	-	10
**20***	15.9 (12 – 16)	-	10
**40***	35.7 (30 – 35)	-	10
**80***	65.2 (62 - 71)	-	10
**Overall PanCK+, CD45-**			
**0**	-	30	-
**15**	11.2 (8 - 13)	3	7
**30**	25.7 (22 – 26)	-	10
**60**	54.4 (46 – 57)	-	10
**120**	117.5 (97 – 112)	-	10
**240**	215.7 (206 - 228)	-	10
**represents proportionate number of spiked cells in marker subset analysis*			

Supplementary Table S14. Analytical Validation: Precision. Recovery of SKBR3 cells spiked into healthy donor blood samples across multiple replicates by 2 independent operators and over multiple days were used to determine the %CV.

A. EpCAM	Low Spike (15 cells)			High Spike (150 cells)			Overall CV%
	Mean	SD	CV%	Mean	SD	CV%	
**Intra-Run**							
User 1	*15.8*	*1.18*	*7.5%*	*150.4*	*3.18*	*2.1%*	***4.8%***
User 2	*15.6*	*1.04*	*6.7%*	*150.6*	*2.83*	*1.9%*	***4.3%***
Cumulative	*15.7*	*0.96*	*6.1%*	*150.5*	*2.67*	*1.8%*	***4.0%***
**Inter-Run**							
User 1	*15.8*	*0.58*	*3.7%*	*150.4*	*0.99*	*0.7%*	***2.2%***
User 2	*15.6*	*0.53*	*3.4%*	*150.6*	*1.4*	*0.9%*	***2.2%***
Cumulative	*15.7*	*0.51*	*3.3%*	*150.5*	*0.6*	*0.4%*	***1.9%***
**Inter-User**							
Inter-User	*15.7*	*0.13*	*0.8%*	*150.5*	*0.11*	*0.1%*	***0.5%***
**OVERALL**	-	-	**7.1%**	-	-	**2.0%**	***4.6%***

B. GATA3	Low Spike (15 cells)			High Spike (150 cells)			Overall CV%
	Mean	SD	CV%	Mean	SD	CV%	
**Intra-Run**							
User 1	15.6	0.95	***6.1%***	151.6	2.45	***1.6%***	***3.9%***
User 2	15.7	0.93	***5.9%***	151.7	2.64	***1.7%***	***3.8%***
Cumulative	15.6	0.85	***5.6%***	151.6	2.34	***1.4%***	***3.5%***
**Inter-Run**							
User 1	15.6	0.41	***2.6%***	151.6	0.58	***0.4%***	***1.5%***
User 2	15.7	0.32	***2.0%***	151.7	0.59	***0.4%***	***1.2%***
Cumulative	15.6	0.19	***1.2%***	151.6	0.41	***0.3%***	***0.8%***
**Inter-User**							
Inter-User	15.6	0.04	***0.2%***	151.6	0.07	***0.0%***	***0.1%***
**OVERALL**	**-**	**-**	***6.0%***	**-**	**-**	1.7%	***3.9%***

C. GCDFP15	Low Spike (15 cells)			High Spike (150 cells)			Overall CV%
	Mean	SD	CV%	Mean	SD	CV%	
**Intra-Run**							
User 1	15.7	0.96	***6.1%***	150.6	2.11	***1.4%***	***3.8%***
User 2	15.7	0.96	***6.1%***	151.3	2.25	***1.5%***	***3.8%***
Cumulative	15.7	0.87	***5.5%***	150.9	2.04	***1.5%***	***3.5%***
**Inter-Run**							
User 1	15.7	0.43	***2.7%***	150.6	0.59	***0.4%***	***1.6%***
User 2	15.7	0.29	***1.9%***	151.3	0.28	***0.2%***	***1.1%***
Cumulative	15.7	0.31	***2.0%***	150.9	0.38	***0.3%***	***1.2%***
**Inter-User**							
Inter-User	15.7	0.03	***0.2%***	150.9	0.49	***0.3%***	***0.3%***
**OVERALL**	-	-	***6.1%***	-	-	***1.5%***	***3.8%***

Supplementary Table S15. Analytical Validation: Guard Banding Studies for Robustness. Guard banding studies established the ability of the Test to not be prone to variations from deliberate controlled variations

Parameter	Range		% Variance	
	Normal	Guard Band (Low, High)	Low	High
**Blood Collection**				
EDTA Vacutainer	Current	New, Near Expiry	4.9%	5.1%
**RBC Lysis**				
Lysis Buffer	5 vol	4 vol, 6 vol	5.9%	4.9%
Incubation Temperature	37°C	35°C, 39°C	2.5%	3.1%
Incubation Time	20 min	15 min, 25 min	2.9%	2.6%
pH	7.2	pH 6.7, pH 7.7	2.1%	1.5%
Centrifugation Speed	400 x *g*	320 x *g*, 480 x *g*	3.1%	2.4%
Centrifugation Temperature	4°C	3.2°C, 4.8°C	2.5%	1.5%
Centrifugation Time	5 min	4 min, 6 min	4.8%	4.1%
**Immunocytochemistry**				
Paraformaldehyde	4%	3.5%, 4.5%	0.9%	2.8%
	20 min	15 min, 25 min	1.3%	2.6%
Triton X-100	0.3%	0.25%, 0.35%	2.0%	2.5%
	25 min	20 min, 30 min	3.5%	1.9%
1° Ab Dilution: Anti-EpCAM	1:500	1:450, 1:550	5.4%	6.7%
1° Ab Dilution: Anti-PanCK	1:500	1:450, 1:550	1.9%	1.7%
1° Ab Dilution: Anti-CD45	1:500	1:450, 1:550	2.1%	3.5%
1° Ab Dilution: Anti-GATA3	1:4	1:3, 1: 5	5.9%	6.6%
1° Ab Dilution: Anti-GCDFP15	1:2	1:1, 1:3	5.3%	4.4%
1° Ab incubation temperature*	25°C	23°C, 27°C	3.3%	2.0%
1° Ab Incubation Time*	60 min	50 min, 70 min	2.4%	3.3%
2° Ab Dilution*	1: 100	1:50, 1: 150	2.5%	2.5%
2° Ab incubation temperature*	25°C	23°C, 27°C	2.6%	2.0%
2° Ab incubation time*	60 min	50 min, 70 min	1.9%	3.7%
**evaluated for GATA3.*				

Supplementary Table S16. Analytical Validation: Impact of Potentially Interfering Substances. The Test was not prone to interference from endogenous agents (deranged serum parameters) and exogenous agents (common non-anticancer drugs)

Agent	Concentration Used	Detected Cells / mL		
		PanCK+, EpCAM+	PanCK+, GCDFP15+	PanCK+, GATA3
Levothyroxine	140 ng / mL	9 (90%)	10 (100%)	9 (90%)
Lisinopril	58 ng / mL	9 (90%)	9 (90%)	9 (90%)
Atorvastatin	30 ng / mL	10 (100%)	9 (90%)	8 (80%)
Metformin	5 μg / mL	10 (100%)	10 (100%)	10 (100%)
Amlodipine	5 ng / mL	9 (90%)	9 (90%)	9 (90%)
Metoprolol	50 ng / mL	8 (80%)	10 (100%)	10 (100%)
Omeprazole	660 ng / mL	9 (90%)	10 (100%)	10 (100%)
Albuterol	4.2 ng / mL	9 (90%)	10 (100%)	9 (90%)
Ranitidine	450 ng / mL	9 (90%)	9 (90%)	10 (100%)
Azithromycin	500 ng / mL	9 (90%)	10 (100%)	10 (100%)
Paracetamol	9.9 μg / mL	9 (90%)	9 (90%)	9 (90%)
Aspirin	3 μg / mL	10 (100%)	10 (100%)	9 (90%)
Loperamide	3.4 ng / mL	9 (90%)	8 (80%)	10 (100%)
Dextromethorphan	2.9 ng / mL	10 (100%)	9 (90%)	10 (100%)
Ulipristal acetate	170 ng / mL	9 (90%)	10 (100%)	9 (90%)
Cholesterol	3 mg / mL	9 (90%)	8 (80%)	8 (80%)
Creatinine	20 μg / mL	8 (80%)	9 (90%)	8 (80%)
Uric Acid	150 μg / mL	9 (90%)	8 (80%)	8 (80%)
Bilirubin	20 μg / mL	9 (90%)	8 (80%)	8 (80%)
Haemoglobin	200 mg / mL	10 (100%)	9 (90%)	8 (80%)
Glucose	3 mg / mL	10 (100%)	9 (90%)	9 (90%)
Control	-	9 (90%)	10 (100%)	10 (100%)
